# C16 Peptide and Ang-1 Improve Functional Disability and Pathological Changes in an Alzheimer’s Disease Model Associated with Vascular Dysfunction

**DOI:** 10.3390/ph15040471

**Published:** 2022-04-13

**Authors:** Xiaoxiao Fu, Jing Wang, Huaying Cai, Hong Jiang, Shu Han

**Affiliations:** 1 Institute of Anatomy, Medical College, Zhejiang University, Hangzhou 310058, China; 21818569@zju.edu.cn; 2 Department of Neurology, Sir Run Run Shaw Hospital, Medical College, Zhejiang University, Hangzhou 310058, China; wangjinjoy@zju.edu.cn (J.W.); caihuaying2004@zju.edu.cn (H.C.); jianghong1975@zju.edu.cn (H.J.)

**Keywords:** Alzheimer’s disease, cerebrovascular dysregulation, inflammation, C16, Ang-1

## Abstract

Alzheimer’s disease (AD) is a neurological disorder characterized by neuronal cell death, tau pathology, and excessive inflammatory responses. Several vascular risk factors contribute to damage of the blood–brain barrier (BBB), secondary leak-out of blood vessels, and infiltration of inflammatory cells, which aggravate the functional disability and pathological changes in AD. Growth factor angiopoietin-1 (Ang-1) can stabilize the endothelium and reduce endothelial permeability by binding to receptor tyrosine kinase 2 (Tie2). C16 peptide (KAFDITYVRLKF) selectively binds to integrin ανβ3 and competitively inhibits leukocyte transmigration into the central nervous system by interfering with leukocyte ligands. In the present study, 45 male Sprague-Dawley (SD) rats were randomly divided into three groups: vehicle group, C16 peptide + Ang1 (C + A) group, and sham control group. The vehicle and C + A groups were subjected to two-vessel occlusion (2-VO) with artery ligation followed by Aβ1-42 injection into the hippocampus. The sham control group underwent sham surgery and injection with an equal amount of phosphate-buffered saline (PBS) instead of Aβ1-42. The C + A group was administered 1 mL of drug containing 2 mg of C16 and 400 µg of Ang-1 daily for 2 weeks. The sham control and vehicle groups were administered 1 mL of PBS for 2 weeks. Our results showed that treatment with Ang-1 plus C16 improved functional disability and reduced neuronal death by inhibiting inflammatory cell infiltration, protecting vascular endothelial cells, and maintaining BBB permeability. The results suggest that these compounds may be potential therapeutic agents for AD and warrant further investigation.

## 1. Introduction

The structural/functional integrity of blood vessels and adequate blood supply are key to maintaining normal brain function [1]. The endothelial monolayer forms the blood–brain barrier (BBB), which maintains cerebrovascular integrity and regulates the transport of most blood cells (e.g., leukocytes), microbial pathogens, and blood-derived macromolecules between the blood and the brain [1]. Neurovascular dysfunction is becoming increasingly recognized in a chronic disorder, Alzheimer’s disease (AD); the vascular risk factors have been shown to promote cognitive decline by acting synergistically with amyloid-β (Aβ) [2,3,4,5]. Brain vascular dysfunction is also implicated in other neuro-inflammatory disorders [4], including multiple sclerosis (MS), stroke, Parkinson’s disease (PD), amyotrophic lateral sclerosis (ALS), traumatic brain injury (TBI), and epilepsy, all of which are associated with the downregulation of tight junction proteins and the activation of endothelial cells [6].

Neuronal damage, a hallmark of neurodegenerative diseases, is associated with chronic activation of innate immune responses in the central nervous system (CNS). The development of novel therapeutic strategies that target pathogenic mechanisms responsible for detrimental inflammation and harness endogenous protective pathways is the key to counteracting neuro-inflammatory diseases [6].

Increased BBB permeability induces edema and inflammatory cell recruitment into target tissues, resulting in nerve demyelination and dysfunction. Growth factor Angiopoietin-1 (Ang-1) binds to the receptor tyrosine kinase 2 (Tie2) to mediate angiogenesis. The binding of Ang-1 to Tie2 stabilizes the endothelium and reduces endothelial permeability, and therefore, is essential to the development of the cardiovascular system [7]. Moreover, a previous study showed that Ang-1 ameliorated inflammation-induced vessel leakage in the CNS and inhibited inflammatory cell infiltration into the spinal cord and the brain in a model of acute experimental allergic encephalomyelitis (EAE) [8].

The accumulation of surface cell adhesion molecules in peripheral blood cells during the course of CNS inflammation is a prerequisite for their crossing of the BBB and their interaction with activated endothelial cells. Integrins are heterodimeric membrane proteins primarily expressed in leukocytes, and serve as adhesion receptors for multiple extracellular matrix components. The integrin ανβ3 is upregulated in autoimmune T cells and has been reported to mediate leukocyte adhesion to the intercellular adhesion molecule-1 (ICAM-1) and facilitate leukocyte migration across the BBB [9]. The synthetic C16 peptide (KAFDITYVRLKF) represents a functional laminin domain that selectively binds to integrin ανβ3 and competitively inhibits leukocyte transmigration into the CNS by interfering with leukocyte ligands [10]. Importantly, C16 is not an immunosuppressant and has no effect on the amount of systemic leukocytes [10].

We investigated the efficacy of a combined treatment of C16 and Ang-1 in a rat model of AD associated with vascular dysfunction. In previous study, we analyzed the gene expression profiles of patients with AD and vascular dementia (VaD) in the Gene Expression Omnibus (GEO) database and found that the expressions of the Ras homolog gene family member A (RhoA), ICAM-1, angiotensinogen (AGT), phosphatidylinositol 3/kinase-protein kinase B (PI3K/AKT), and signal transducer and activator of transcription 3 (STAT3) were upregulated in both patient groups compared with healthy controls. The potential mechanisms underlying the upregulation of these genes were also investigated. Our data provide new insights into targeting the inflammatory microenvironment of the CNS to treat neurodegenerative diseases.

## 2. Results

### 2.1. Treatment with C16 Plus Ang-1 Alleviated Memory Impairment in AD Rats with Vascular Dysfunction

**Figure 1:**
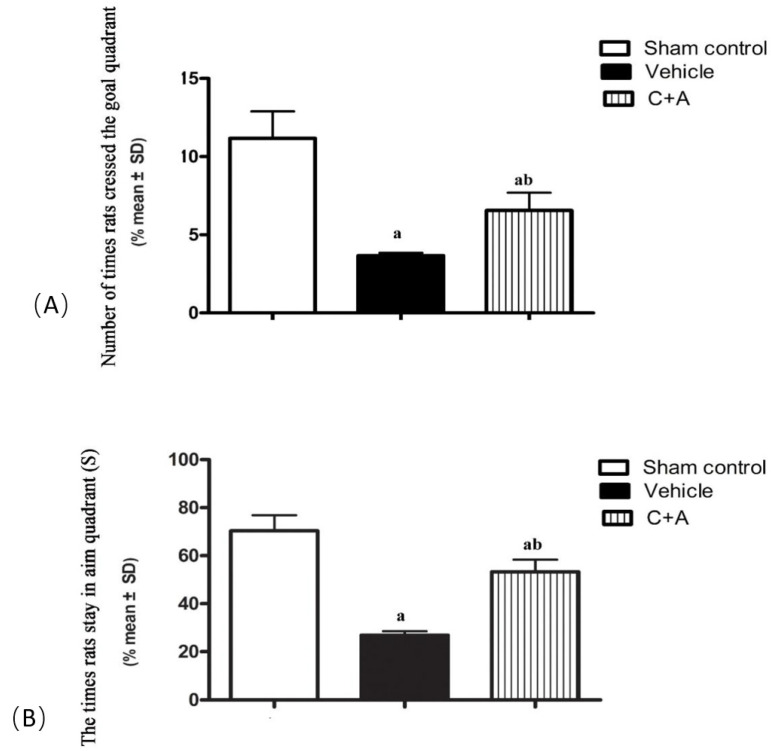
The results of the space exploration test showed that the number of times that rats crossed the platform quadrant (**A**) and the average time (s) that animals stayed in the platform quadrant (**B**) in the vehicle group were significantly decreased compared with the sham control. This phenomenon was effectively reversed by treatment with C16 plus Ang-1. a, *p* < 0.05 vs. sham control group; b, *p* < 0.05 vs. vehicle group.

The average escape time of vehicle rats was markedly shorter compared with sham controls (*p* < 0.05; Table 1). Rats treated with C16 plus Ang-1 showed significantly improved escape performance compared with the vehicle group (Table 1). Vehicle rats also failed to find the hidden platform during the acquisition phase (*p* < 0.05, Figure 1A,B), suggesting impaired spatial memory in this group. In the hidden platform task, vehicle rats swam more slowly and crossed the target quadrant fewer times compared to the C + A group (*p* < 0.05, Figure 1A,B). However, the combined treatment with C16 plus Ang-1 increased the time that animals spent in the goal quadrant.

### 2.2. Treatment with C16 Plus Ang-1 Suppressed Inflammation in the CNS

**Figure 2:**
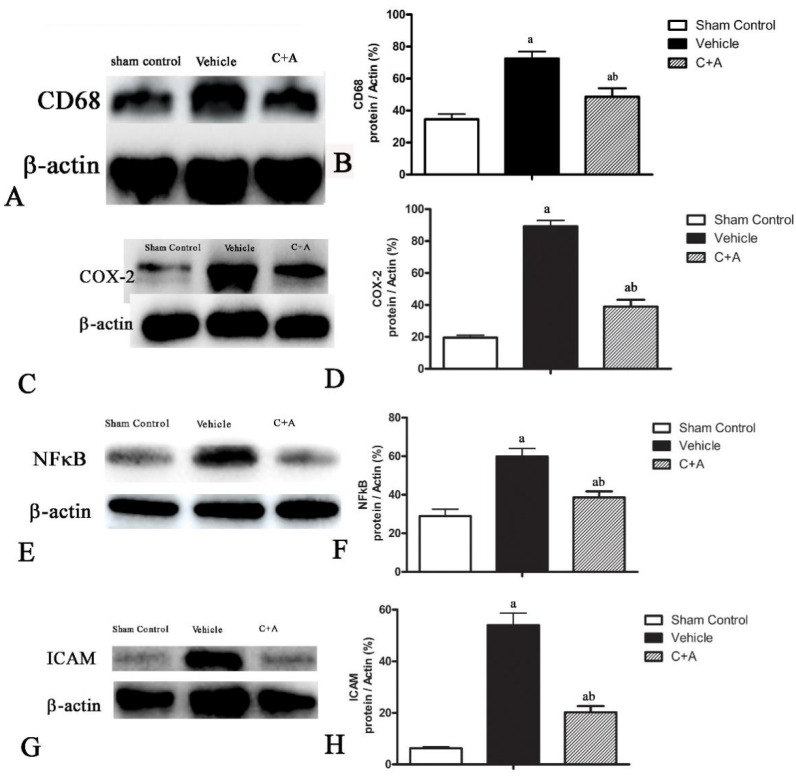
Western blot analysis of the expressions of (**A**,**B**) CD68/ED1, (**C**,**D**) cyclooxygenase-2 (COX-2), (**E**,**F**) nuclear factor kappa B (NF-κB), (**G**,**H**) intercellular cell adhesion molecule-1 (ICAM-1 in the sham control, vehicle, and C + A groups (**B**,**D**,**F**,**H**). Semi-quantitative profiles of protein bands showed that C16 plus Ang-1 suppressed the upregulation of CD68, COX-2, NF-κB, and ICAM-1 in the vehicle group. a, *p* < 0.05 vs. sham control group; b, *p* < 0.05 vs. vehicle group. The molecular weight (MW): CD68, 100 KD; COX-2, 75 KD; NF-κB, 65 KD; ICAM-1, 92 KD; β-actin, 42 KD.

**Figure 3:**
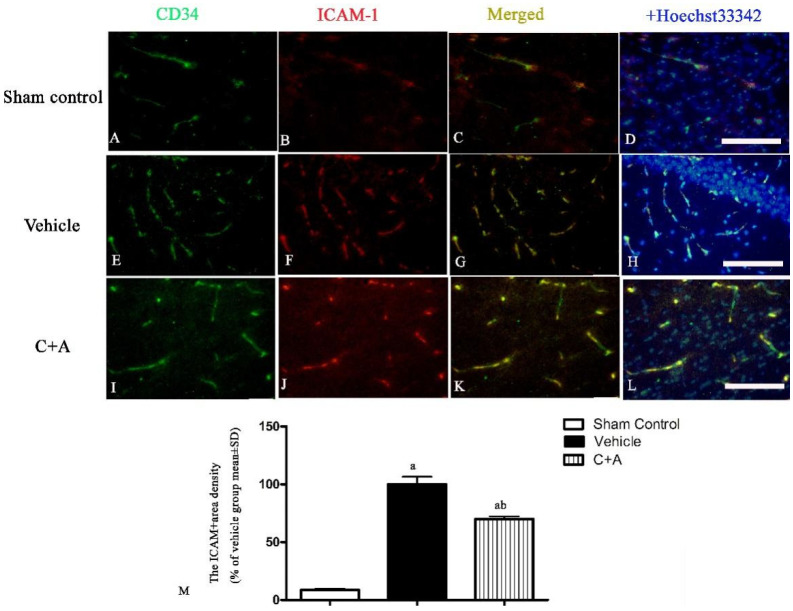
(**A**–**L**): Double immunofluorescence staining of microvessel marker CD34 (green) and ICAM-1 (red) in all groups. The nuclei were visualized by Hoechst 33342 staining (blue). (**M**) Quantitation of ICAM-1-labeled microvessels showed that treatment with C16 plus Ang-1 reduced the amount of ICAM-1-labeled vessels in AD rats. Scale bar = 100 µm. a, *p* < 0.05 vs. sham control group; b, *p* < 0.05 vs. vehicle group.

**Figure 4:**
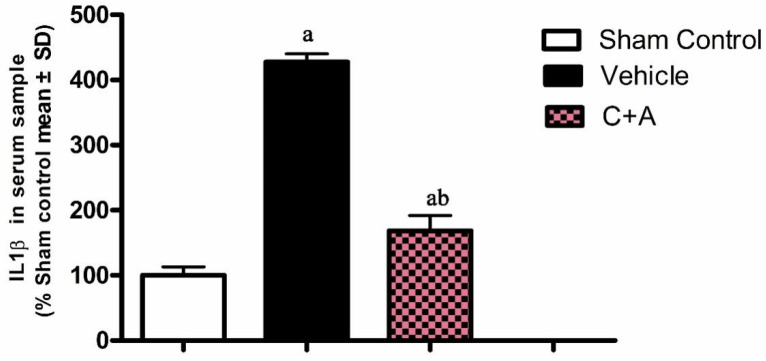
ELISA results showed that treatment with C16 plus Ang-1 decreased the serum level of pro-inflammatory factor IL-1β. a, *p* < 0.05 vs. sham control group; b, *p* < 0.05 vs. vehicle group.

The expressions of macrophage-specific marker CD68 (Figure 2A,B), oxide stress-related factor Cox-2 (Figure 2C,D) and pro-inflammatory cytokine NF-κB (Figure 2E,F) were upregulated in the vehicle group, while treatment with C16 plus Ang-1 effectively suppressed inflammation-related gene expression (Figure 2A–F). ICAM-1, which enables leukocytes to effectively migrate across the endothelium, was upregulated in vehicle-treated animals, and this phenomenon was effectively reversed by treatment with C16 plus Ang-1 (Figure 2G,H). The results of immunofluorescence staining also confirmed the changes in the expression of CD68 (Supplementary Figure S1) and ICAM-1 (Figure 3). Moreover, compared to the sham control group, the concentration of pro-inflammatory cytokine IL-1β was significantly elevated in rats subjected to 2-VO operation and Aβ1-42 injection (Figure 4). C16 plus Ang-1, however, significantly inhibited the upregulation of IL-1β in AD rats (Figure 4).

### 2.3. Treatment with C16 Plus Ang-1 Reduced BBB Permeability and Blood Vessel Leakage

**Figure 5:**
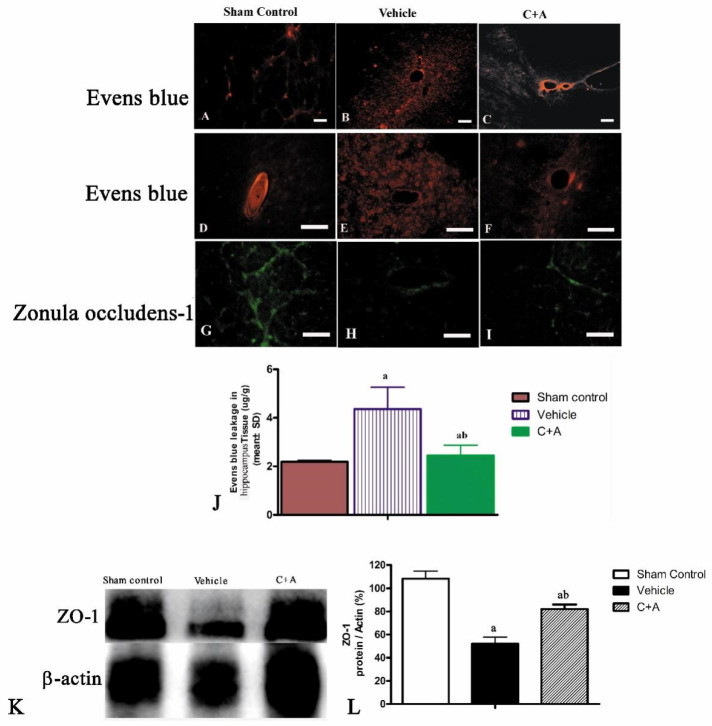
(**A**–**F**): Images of Evans blue (a dye to detect blood vessel leakage) staining (red) of the blood–brain barrier (BBB) in the (**A**,**D**) sham control, (**B**,**E**) vehicle, and (**C**,**F**) C + A groups. Increased blood vessel leakage in the vehicle group, as indicated by the amount of Evans blue dye that leaked out into surrounding blood vessels, was alleviated by the C + A treatment. (**G**–**I**): Immunofluorescence staining images (green) showed that ZO-1 (a marker of tight junctions) in micro-blood vessels was downregulated in the vehicle group, but was restored by C16 + Ang-1 treatment. Scale bar = 100 µm. (**J**) Semi-quantitative profiles of Evans blue staining. (**K**) Western blot analysis of the expression of ZO-1. (**L**) Semi-quantitative profiles of protein bands showed that C16 plus Ang-1 increased the expression of ZO-1 in the vehicle group. ZO-1, 240 KD; β-actin, 42 KD. a, *p* < 0.05 vs. sham control group; b, *p* < 0.05 vs. vehicle group. a, *p* < 0.05 vs. sham control group; b, *p* < 0.05 vs. vehicle group.

Evans blue dye extravasation is indicative of edema and compromised blood vessel integrity in tissues. The vehicle group showed severe vasculature leakage (Figure 5B,E), while the C + A group (Figure 5C,F) showed markedly less leakage from surrounding blood vessels (Figure 5J). Furthermore, ZO-1, a specific marker of tight junctions between endothelial cells in blood vessels, was downregulated in AD rats, but was restored by treatment with C16 plus Ang-1 (Figure 5G–I,K,L).

### 2.4. Treatment with C16 Plus Ang-1 Reduced Autophagy and Neuronal Apoptosis, Restored the Expression of ACH and CHAT, Alleviated Sn Loss in AD Rats

**Figure 6:**
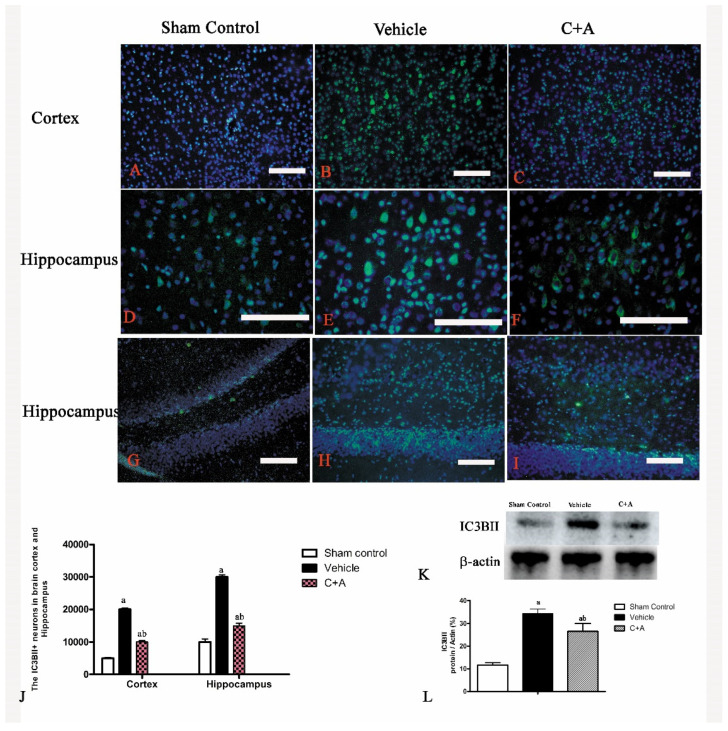
(**A**–**I**): Immunofluorescence staining (green) of LC3BII in the motor cortex and hippocampus. Scale bar = 100 µm. (**J**) The quantification of LC3BII-positive cells showed that treatment with C16 plus Ang-1 reduced the number of LC3BII-positive cells compared to the vehicle controls. (**K**) Western blot analysis of the expressions of autophagy marker LC3BII. (**L**) Semi-quantitative profiles of protein bands showed that C16 plus Ang-1 suppressed the upregulation of LC3BII in the vehicle group. LC3BII, 14.16 KD; β-actin, 42 KD. a, *p* < 0.05 vs. sham control group; b, *p* < 0.05 vs. vehicle group.

The injection of C16 plus Ang-1 decreased the cytoplasmic level of LC3BII, an autophagy marker, in both the cortex and hippocampus of AD rats (Figure 6). Moreover, the level of active caspase-3, an enzyme involved in mammalian cell apoptosis, as well as LC3BII, was increased in the vehicle group but was downregulated by the C16 + Ang-1 treatment (Figure 6A–L).

**Figure 7:**
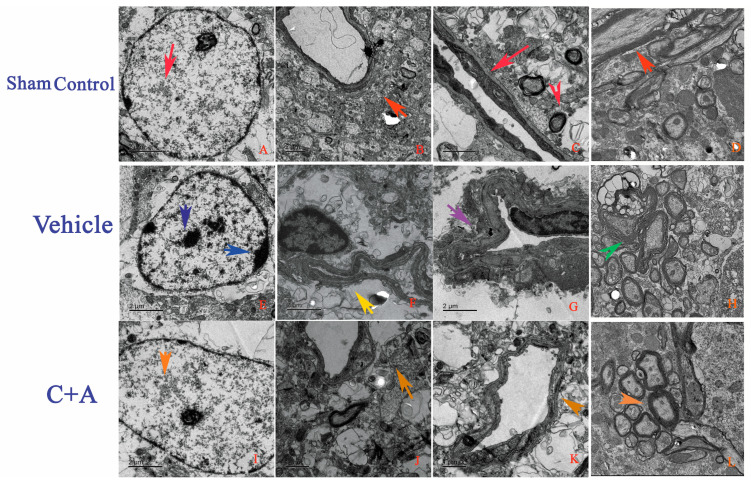
Electron micrographs show the ultrastructural morphology of the (**A**–**D**) sham control, (**E**–**H**) vehicle, and (**I**–**L**) C + A groups. The sham control rats showed (**A**) neuronal nuclei with uncondensed chromatin (red arrow in (**A**)). There was (**B**) no tissue edema or blood vessel leakage (red arrow in (**B**)). Intact tight junctions (red arrow in (**C**)) and myelinated axons surrounded by dark, ring-shaped myelin sheaths were also observed (red arrows in (**C**,**D**)). In the vehicle group, (**E**) neuronal apoptosis was evidenced by shrunken nuclei with marginated, fragmented, and condensed nuclear chromatin (showed by two arrows in (**E**)). (**F**) Severe blood vessel leakage and tissue edema in the extracellular space surrounding the vessels (yellow arrow in (**F**)). (**G**) Loosening of tight junctions between the endothelium (purple arrow in (**G**)). (**H**) Myelin sheath loosening and splitting (green arrow in (**H**)). Treatment with C16 plus Ang-1 reduced morphological changes of the nuclei (orange arrow in (**I**)), alleviated perivascular edema (orange arrow in (**J**)), prevented destruction of tight junctions (orange arrow in (**K**)), and decreased myelin sheath splitting (orange arrow in (**L**)).

The ultrastructural morphology of the hippocampus and the cortex of the sham control (Figure 7A–D), vehicle (Figure 7E–H), and C + A (Figure 7I–L) groups were examined using transmission electron microscopy. The sham control group showed neuronal nuclei with uncondensed chromatin and intact tight junctions, and exhibited no blood vessel leakage or tissue edema. The myelinated axons were also surrounded by dark, ring-shaped myelin sheaths (Figure 7A–D). Neuronal apoptosis was found in vehicle animals, as indicated by shrunken nuclei with marginated, condensed, and fragmented nuclear chromatin, tissue edema in the extracellular space surrounding the vessels, blood vessel leakage, and the loosening of the tight junction between the endothelium. The loosening and splitting of myelin sheaths were also observed (Figure 7E–H). In the C + A group, the morphology of the nuclei was relatively normal. Destruction of tight junctions, perivascular edema, and myelin sheath splitting were also alleviated (Figure 7I–L).

**Figure 8:**
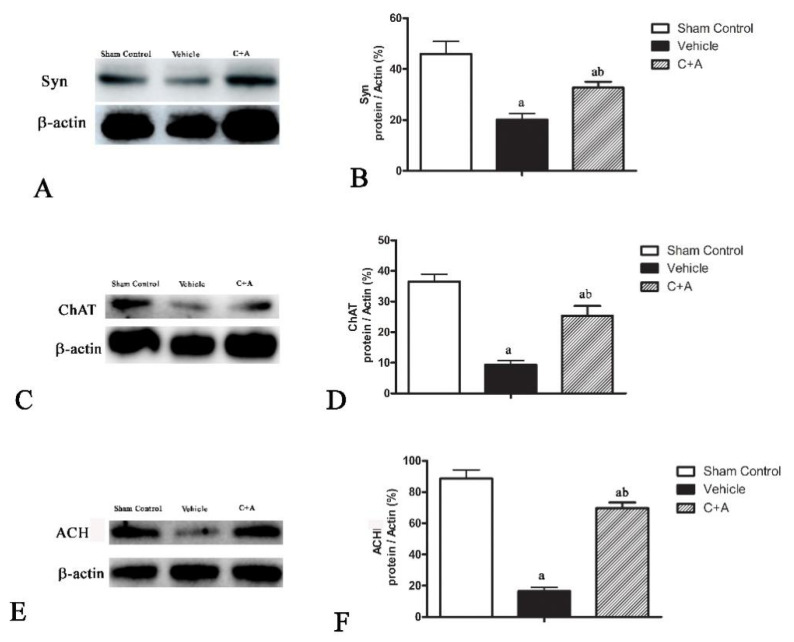
Western blot analysis of the expression of (**A**,**B**) synaptophysin (Syn), (**C**,**D**) choline acetyltransferase (CHAT), and (**E**,**F**) acetylcholine (ACH) in all three experimental groups. (**B**,**D**,**F**) Semi-quantitative profiles of protein bands showed that the C16 + Ang-1 treatment restored the downregulation of Syn, CHAT, and ACH in the vehicle group. a, *p* < 0.05 vs. sham control group; b, *p* < 0.05 vs. vehicle group. Molecular weight (MW): Syn, 38 KD; CHAT, 83 KD; ACH, 146KD; β-actin, 42 KD.

**Figure 9:**
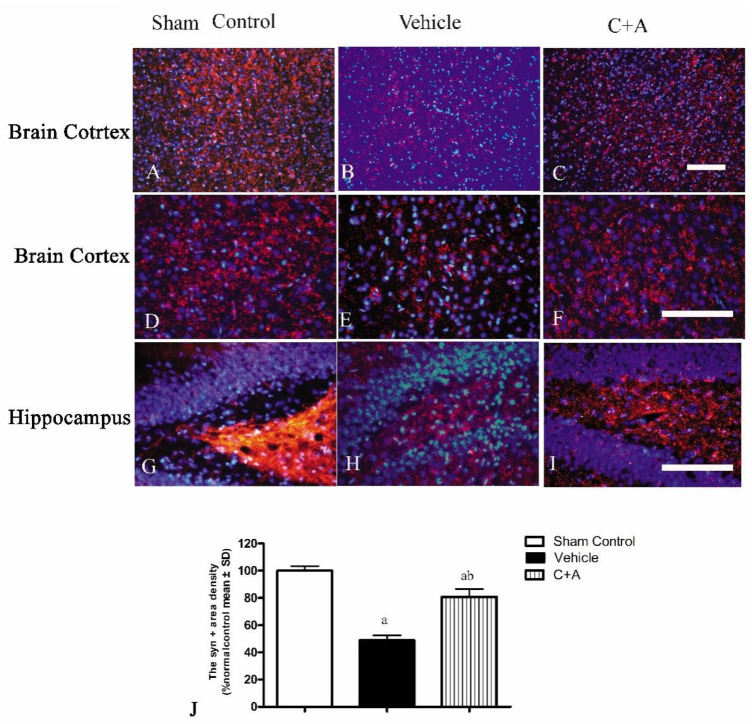
(**A**–**I**): Immunofluorescence staining (red) of Syn in the motor cortex (**A**–**F**) and hippocampus (**G**–**I**). The nuclei of neurons in the cortex and hippocampus were visualized by Hoechst 33342 staining (blue). (**J**): Quantification of Syn-positive areas showed that treatment with C16 plus Ang-1 induced the expression of Syn in AD rats. Scale bar = 100 µm. a, *p* < 0.05 vs. sham control group; b, *p* < 0.05 vs. vehicle group.

**Figure 10:**
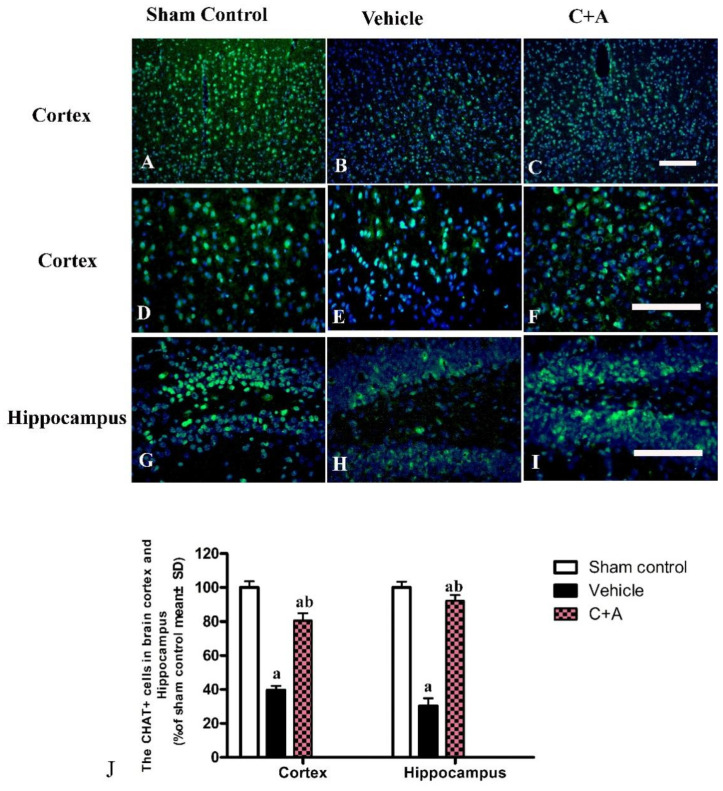
(**A**–**I**): Immunofluorescence staining (green) of CHAT in the motor cortex (**A**–**F**) and hippocampus (**G**–**I**). The nuclei of neurons in the cortex and hippocampus were visualized by Hoechst 33342 staining (blue). (**J**): Quantification of CHAT-positive cells indicated that treatment with C16 plus Ang-1 induced the expression of CHAT in AD rats. Scale bar = 100 µm. a, *p* < 0.05 vs. sham control group; b, *p* < 0.05 vs. vehicle group.

**Figure 11:**
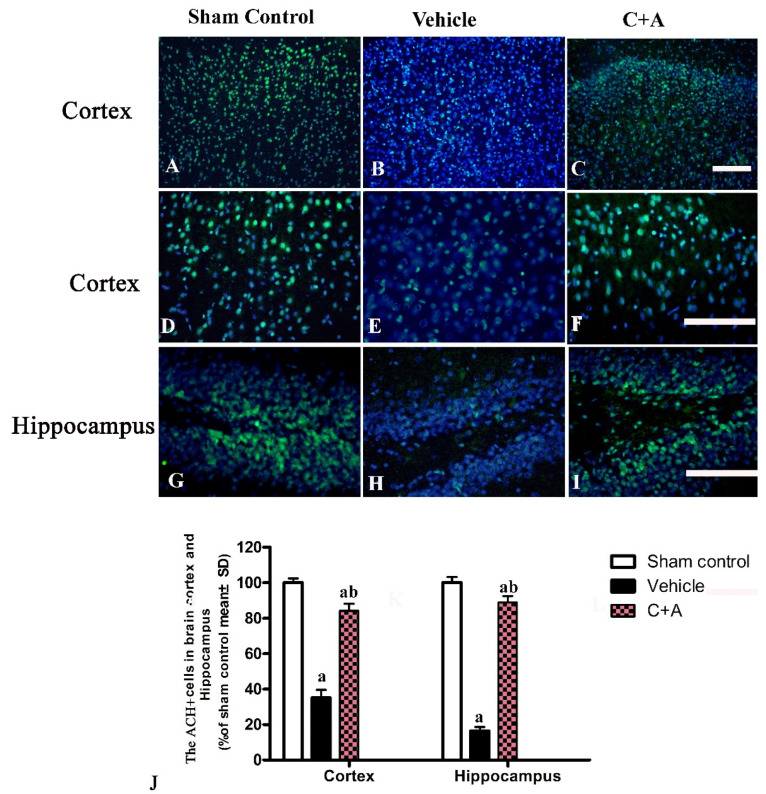
(**A**–**I**): Immunofluorescence staining (green) of ACH in the motor cortex (**A**–**F**) and hippocampus (**G**–**I**). The nuclei of neurons in the cortex and hippocampus were visualized by Hoechst 33342 staining (blue). (**J**): Quantification of ACH-positive cells showed that treatment with C16 plus Ang-1 induced the expression of ACH in AD rats. Scale bar = 100 µm. a, *p* < 0.05 vs. sham control group; b, *p* < 0.5 vs. vehicle group.

The levels of Syn (Figure 8A,B), CHAT (Figure 8C,D), and ACH (Figure 8E,F) in vehicle rats were decreased compared to those in the sham control group, while the C16 + Ang-1 treatment restored their expressions, suggesting that this treatment not only maintained the number of synapses, but also protected cholinergic neurons. Furthermore, the immunofluorescence staining also confirmed the changes in the expressions of Syn (Figure 9), CHAT (Figure 10), and ACH (Figure 11) in each group.

### 2.5. Treatment with C16 Plus Ang-1 Affected the Signaling Pathway Related to Inflammation

**Figure 12:**
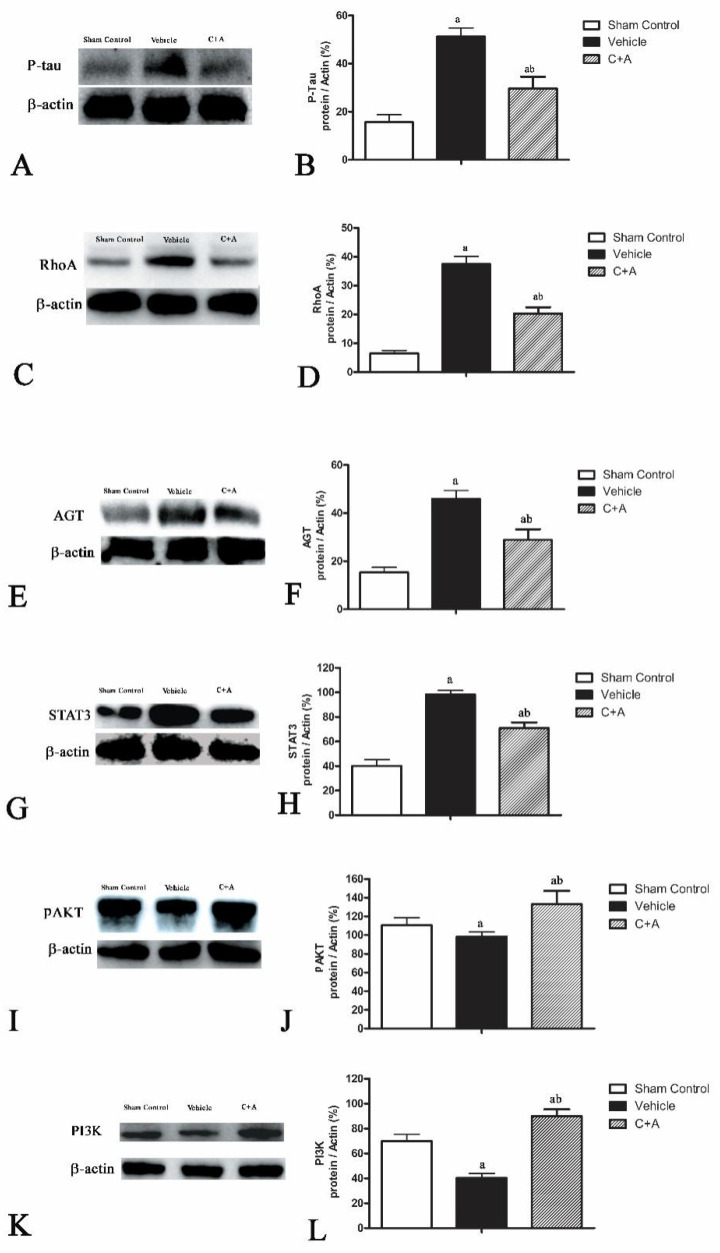
Western blot analysis of the level of (**A**,**B**) phosphorylated tau (p-tau), (**C**,**D**) RhoA/Rho kinase, (**E**,**F**) angiotensinogen (AGT), (**G**,**H**) signal transducers and activators of transcription 3 (STAT), (**I**,**J**) phosphorylated AKT (p-AKT), and (**K**,**L**) phospho-Tyr467 (PI3K/P85) in all groups. (**B**,**D**,**F**,**H**,**J**,**L**) Semi-quantitative profiles of protein bands showed that treatment with C16 plus Ang-1 suppressed the upregulation of p-tau, RhoA, AGT, and STAT3 in AD rats, but increased the expression of p-AKT and PI3K. a, *p* < 0.05 vs. sham control group; b, *p* < 0.05 vs. vehicle group. Molecular weight (MW): p-tau, 68 KD; RhoA, 22 KD; AGT, 52 KD; STAT3, 88 KD; p-AKT, 60 KD; PI3K, 85 KD; β-actin, 42 KD.

**Figure 13:**
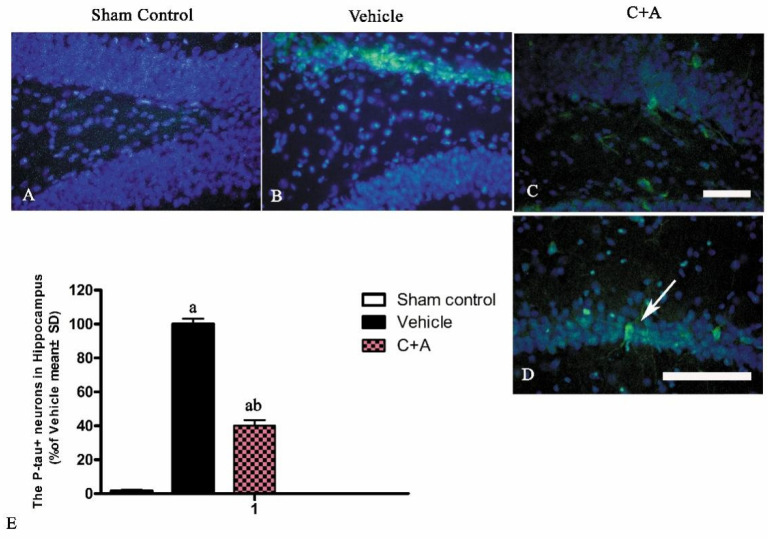
(**A**–**D**) Immunofluorescence staining (green) of p-tau in the hippocampus. The white arrow in (**D**) indicates a neuron expressing p-tau. The cell nuclei in the cortex and hippocampus were visualized by Hoechst 33342 staining (blue). (**E**) Quantification of p-tau-positive cells showed that treatment with C16 plus Ang-1 reduced the expression of p-tau in AD rats. Scale bar = 100 µm. a, *p* < 0.05 vs. sham control group; b, *p* < 0.05 vs. vehicle group.

Western blot analysis and immunofluorescence staining showed that elevated expressions of p-tau (Figure 12A,B and Figure 13), RhoA (Figure 12C,D; Supplementary Figure S3), AGT (Figure 12E,F; Supplementary Figure S4) and STAT3 (Figure 12G,H; Supplementary Figure S5) in AD rats were downregulated by treatment with C16 plus Ang-1.

### 2.6. The Activity of C16 Plus Ang1 in AD Is Mediated by the PI3K/Akt Pathway

Lastly, we measured the level of p85, a regulatory subunit of PI3K. Treatment with C16 plus Ang1 significantly induced the phosphorylation of AKT (Figure 12I,J), compared with the vehicle and sham control groups (Supplementary Figure S6A–G). Additionally, C16 plus Ang1 significantly upregulated the expression of PI3K/p85 (Figure 12K,L; Supplementary Figure S6H–N). These results indicate that the activity of C16 plus Ang1 was mediated by the PI3K/Akt pathway.

## 3. Discussion

The CNS is an “immuno-privileged” organ that hosts defensive barriers, such as the choroid plexus, the perivascular space, and the meninges [11]. A coordinated response between microglia and other CNS cells is involved in neuro-inflammation, such as the infiltration of peripheral immune cells and astrocytes into the CNS. Acute neuro-inflammation, characterized by microgliosis and the release of inflammatory mediators, can be elicited by different types of stimuli, including ischemia, trauma, toxins, and infections [11]. Neuro-inflammatory responses in AD are complex [6]. Previous studies have shown the strong contribution of inflammation to the pathological process of AD and have reported a reduced risk of developing AD for people who had been treated with anti-inflammatory drugs before the onset of the disease [6].

Neurodegenerative and neuro-inflammatory diseases are characterized by the recruitment of circulating innate and adaptive immune cells in the CNS. Neuro-inflammatory responses, ranging from glial cell activation to immune cell infiltration, are frequently associated with the pathogenesis of AD [12]. During neuro-inflammatory and neurodegenerative disorders, the protective function of the BBB may be severely impaired, resulting in detrimental neuro-inflammatory changes. Infiltrated leukocytes sustain detrimental responses in the CNS by releasing pro-inflammatory cytokines to aggravate BBB damage, glial cell activation, and neural cell death [13].

Our previous studies of C16 [9] and Ang-1 [10] showed that C16 peptide rescued blood vessels by acting as an agonist, and by binding only to ανβ3 and α5β1 integrin, two well-known factors that promote the survival of endothelial cells. Moreover, Ang-1, acting as a growth factor of vascular endothelial cells, protects micro-blood vessels, reduces blood–brain barrier (BBB) permeability, and prevents blood vessel leakage. Even though they may share the same mechanism, we assumed that Ang-1 and C16 might gain better synergistic effects through targeting different pathways.

Many factors are implicated in the changes of BBB permeability, such as matrix metalloproteinases, free radicals, and inflammatory cytokines/chemokines, which may lead to brain tissue damage during the course of cerebral ischemia [14]. Chemokines are a subgroup of cytokines initially described as “chemoattractant”, and have been shown to induce leukocyte chemotaxis, thereby increasing BBB permeability and impairing the integrity thereof [15].

In our study, 2-VO operation followed by Aβ1-42 injection effectively destroyed the integrity of the BBB and induced neuro-inflammatory responses. The spatial memory decline, neuronal death in the bilateral hippocampus, increased BBB permeability and upregulated pro-inflammatory factors, as well as extensive inflammatory cell infiltration into the brain parenchyma confirmed that the AD model was successfully established.

Tauopathies are characterized by neuro-inflammation and intracellular accumulation of neurofibrillary tangles, in which hyper-phosphorylated tau (abnormal phosphorylation of tau) protein aggregates [15]. Our results showed that hyper-phosphorylated tau was upregulated in hippocampal neurons of the vehicle group compared with sham control rats, while this phenomenon was significantly attenuated by the C16 + Ang-1 treatment. Since accumulation of microtubule-associated protein tau within hippocampal neurons is related to multiple functional impairments, these findings further confirmed the protective effects of these compounds against memory impairment.

Cholinergic transmission is critical to high-order brain functions, such as memory, learning, and attention. Alzheimer’s disease (AD) is characterized by cognitive decline associated with a specific degeneration of cholinergic neurons. A decrease in choline acetyltransferase (ChAT) enzymatic activity indicates the loss of cholinergic neurons [16]. Moreover, synapse-associated proteins, especially presynaptic Syn, can promote synaptic plasticity. Syn deficiency is correlated with cognitive decline in aging-related neurodegenerative disorders [17]. In the current study, the combination of C16 and Ang-1 alleviated AD-induced Syn reduction, indicating improved cognitive function in the drug-treated groups.

In the present study, we found that the combined use of C16 and Ang-1 exhibited potentially lasting effects. The intravenous injection of C16 and Ang-1 improved vascular-related outcomes (i.e., endothelial cell death and dysfunction, leukocyte extravasation, etc.). As Ang-1 only acts through Tie2, a tyrosine kinase receptor mainly expressed by endothelial cells, Ang-1 treatment mainly reduced the loss of blood vessels, and thus alleviated ischemia, prevented tissue loss, and improved memory, all of which were correlated with the amount of rescued blood vessels [10]. Moreover, integrins play a key role in the leukocyte accumulation and adhesion process by specifically binding to ανβ3. C16 competitively inhibits the transmigration of leukocytes when they cross the endothelium. Inflammatory cell infiltration activates a number of noxious factors that may contribute to secondary injuries. Therefore, treatment with C16 plus Ang-1 suppressed secondary injuries and prevented further tissue damage in AD rats. Less abnormal ultrastructure observed in electron microscopy further confirmed the positive effects.

Our previous study also identified some upregulated genes in both AD and VaD patients, including RhoA, ICAM, AGT, and STAT3, all of which are related to the inflammatory microenvironment in the CNS and could be downregulated by anti-inflammatory therapeutic compounds.

The RhoA/Rho-associated protein kinase pathway plays an important role in regulating cell migration [18]. Angiotensinogen (AGT) is a unique precursor of all angiotensin peptides [19]. AGT is related to cardiovascular diseases, and the expression of AGT can be adjusted by the Rho-associated protein kinase pathway [20].

ICAM-1 is essential for the capture and migration of leucocytes [21]. Inflammation is a key driver of tissue destruction in ischemia reperfusion injury. IL-1β promotes inflammation in various autoimmune and inflammatory diseases [22]. The renin–angiotensin system also contributes to brain damage and cognitive decline caused by chronic cerebral ischemia [23]. A previous study revealed that chronic cerebral hypo-perfusion significantly increased renin activity in activated astrocytes and micro-vessels, and induced angiotensinogen expression in white matter. Thus, the inhibition of angiotensinogen might be a promising therapeutic option for subcortical vascular dementia [24].

Reactive astrogliosis is also a hallmark of AD. The transcription factor STAT3 is a canonical inducer of astrogliosis and has been reported to be activated in an AD mouse model. The STAT3-deficient APP/PS1 mice showed a decreased Aβ level and plaque burden, and were largely protected from neuronal network imbalance [25]. STAT3 deletion in astrocytes also greatly ameliorated memory decline and improved spatial learning in APP/PS1 mice, suggesting STAT3-mediated astrogliosis as a promising therapeutic target in AD [25].

The PI3K/Akt signaling pathway is involved in the antioxidant, neuroprotective, and anti-inflammatory effects of propofol, a widely used anesthetic agent that can attenuate subarachnoid hemorrhage-induced early brain injury via inhibiting oxidative reactions and inflammation [26]. The NF-κB/COX-2/tumor necrosis factor alpha (TNF-α)/IL-1β inflammatory pathway has been reported to positively mediate inflammation in the brain, while the PI3K/Akt signaling pathway negatively regulates inflammatory responses [27] and oxidative stress [28]. Here, treatment with C16 plus Ang-1 upregulated p85 and p-AKT and suppressed the NF-κB/COX-2/TNF-α/IL-1β inflammatory pathway compared with the vehicle animals, implying that brain inflammation in AD possibly acts through activation of the PI3K/Akt pathway.

Microglial activation (MA), a key feature of AD, has been considered a major contributor to progressive neuronal injury by releasing neurotoxic products. Microglial cells undergo two types of activation, each of which acquires a neurotoxic phenotype (M1-like) or a neuroprotective phenotype (M2-like). M1-like microglia build up a detrimental microenvironment for neurons by secreting reactive oxygen species (ROS) and inflammatory cytokines. By contrast, M2-like microglia produce anti-inflammatory mediators and neurotrophic factors, thereby generating a supportive microenvironment for neurons. The activation of the M1/M2 phenotype plays an essential role in AD, and the balance of the M1/M2 phenotype is regulated by the PI3K/AKT pathway. Akt downregulation and NF-κB upregulation have been observed in microglial cells after Aβ42 incubation. Moreover, LY294002, an Akt inhibitor, has been reported to enhance the expression of M1 marker [29]. The above evidence suggests a close relationship between MA and PI3K/AKT signaling. In a recent in vitro study, we explored the involvement of the PI3K/AKT pathway in the anti-MA activity of Ang1 plus C16. The results showed that C16 plus Ang1 significantly upregulated Tie2, which was markedly downregulated by PI3K/AKT inhibitors and the anti-Tie2 antibody [30]. Furthermore, treatment with Ang1 plus C16 significantly induced the expression of p85 and p-AKT, and this phenomenon was downregulated by the inhibition of α5β1 integrin, ανβ3 integrin, PI3K, AKT, and Tie2 [30]. These findings indicate that the anti-MA activity of C16 plus Ang1 was mediated by Tie2, ανβ3 and/or α5β1 integrins, as well as the PI3K/AKT pathway [30]. In the present study, the number of CD68/ED1 (a marker of activated microglia)-positive cells in the C + A group was notably lower compared to the vehicle group, which is consistent with our previous study. Thus, C16 plus Ang-1 not only reduced immune cell infiltration into the CNS but also inhibited the activation of glial cells, both of which were associated with the pathogenesis of AD and vascular dysfunction.

Although the therapeutic effects of C16 combined with Ang-1 have been confirmed, the side effects and toxicity at high doses remain unclear. The dose adjustment for this combined therapy is ongoing in our laboratory.

## 4. Materials and Methods

### 4.1. Animals

Forty-five male SPF SD rats (age: 10–12 weeks; weight: 250 ± 30 g) were purchased from the Shanghai SIPPR-BK Laboratory Animal Center of the Chinese Academy of Sciences. All rats were housed at a constant room temperature (20 ± 2 °C), supplied with sterilized water and food, and acclimated for a week prior to experiments. The experimental protocols were approved by the Ethics Committee of Zhejiang University Medical College (SRRSH202102016).

Rats were randomly assigned into three groups (*n* = 15 per group): the vehicle (2-VO + Aβ1-42 + PBS) group, the C + A (2-VO + Aβ1-42 + C16 + Ang-1) group, and the sham control group. The vehicle and the C + A groups were subjected to two-vessel occlusion (2-VO) followed by Aβ1-42 injection into the hippocampus. Rats in the sham control group underwent the same procedure of 2-VO without artery ligation. Further, instead of Aβ1-42, sham control rats were injected with an equal amount of PBS. The C + A group was administered 1 mL of drug containing 2 mg of C16 and 400 µg of Ang-1 daily for 2 weeks via intravenous tail vein injection. The vehicle and sham control groups were administered 1 mL of PBS for 2 weeks. The flowchart of the study is shown in Supplementary Figure S7.

For each group, 11 out of 15 rats were intracardially perfused with cold saline, followed by 4% paraformaldehyde. Then, 5 (one was infused with EB dye) of the 11 brains were fixed in 4% paraformaldehyde and subsequently soaked in 30% sucrose to make frozen sections; 3 of the 11 brains were fixed in 2.5% glutaraldehyde solution and immersed in 1% osmium tetroxide to make transmission electron sections; another 3 of the 11 brains were dehydrated with alcohol, isopropanol, and n-butanol to make paraffin-embedded sections for the TUNEL assay. Five sections (three visual fields per slide) of each brain were randomly selected for staining and analysis. The remaining 4 rats were sacrificed by decapitation; their blood samples were collected for ELISA and the fresh tissues were used for Western blot analysis.

### 4.2. 2-VO

Rats were anesthetized with 3% pentobarbital sodium (1.5 mL/kg body weight; 922L038, Shanghai Curie biological science and Technology), and then, the hair on the anterior neck was shaved. The target animal was placed in the supine position and disinfected with iodophor (131101 A, ShanDong LIRCON Medical, De Zhou, Shandong, china). Next, a midline cervical incision was made; the fascia was cut, the sternocleidomastoid muscles were separated, and the bilateral common carotid arteries were exposed. A double ligation was performed between the distal and proximal ends using a 5-0 silk thread. Then, the incision was sprayed with penicillin (F4046203, North China Pharmaceutical Group), and the skin was sutured. The body temperature of the rat was maintained between 36.5 and 37.5 °C [30]. The mortality rate following 2-VO operation was 20%. Most animals died 1 day after the operation. The deceased animals were added up immediately. If the animal survived the 2-VO operation for 1 week, it was subjected to Aβ1-42 injection and further analysis.

### 4.3. Aβ1-42 Injection

Aβ1-42 (Millipore, Billerica, MA, USA) was dissolved in 1% NH_3_·H_2_O to prepare a solution with a concentration of 1 μg/μL and incubated for 5 days at 37 °C. At 1 week after the 2-VO operation, rats in the vehicle and C + A groups were anesthetized via an intraperitoneal injection of sodium pentobarbital (60 mg/kg) and then placed in a stereotaxic frame (Ambala, Haryana, India). An incision was made in the dorso-ventral region at 3.6 mm (a site located at 4 mm posterior and 3 mm medio-lateral to bregma) [30]. After 72 h, 1.0 µL of mature Aβ1-42 was injected bilaterally into the hippocampus using a 1-µL Hamilton syringe at a rate of 0.1 µL/min. The sham control group was injected with an equal amount of PBS. The needle was left in place for 5 min to allow complete diffusion of the solution. A small amount of bone wax was used to seal the bone. After being sprayed with penicillin, the skin was sutured. The body temperature of the rat was maintained between 36.5 and 37 °C throughout the operation.

### 4.4. Behavioral Test

The Morris water maze (Smart-Mass, Panlab, Barcelona, Spain) test was performed, including the place navigation test and the space exploration experiment.

The place navigation test assesses memory performance and spatial learning. One day before grouping, animals were placed in water for 120 s to become familiar with the environment. The formal experiment began 3 days after modeling and lasted for 6 days. Rats were trained twice a day (morning and afternoon). The target animal was placed in a pool and released facing the sidewall in each trial. The starting points were the same for all rats in the same training session. The average time that each group spent was measured to determine the escape latency, with an upper limit of 120 s. If the animal failed to find the platform within 120 s, it was guided to the platform and allowed to stay there for 10 s to strengthen the memory. The escape latency of these animals was recorded as 120 s.

On day 7, the space exploration experiment was performed to assess long-term spatial memory. The platform was first removed from the water environment. The number of times that the rat crossed the area where the platform was located and the average time that the animal stayed in the target quadrant were recorded. The test was performed by investigators who were blinded to the treatments of the animals.

### 4.5. Perfusion and Tissue Processing

At 4 weeks after Aβ1-42 injection, animals were anesthetized with sodium pentobarbital and perfused intracardially with cold saline, followed by 4% paraformaldehyde. The brain was harvested from each animal, fixed in 4% paraformaldehyde for 4 h and then soaked in 30% sucrose. Coronal sections (20 um) were obtained using a Leica cryostat and a freezing microtome (Leica, Buffalo Grove, IL, USA). The sections were mounted onto 0.02% poly-L-lysine-coated slides and subjected to immunofluorescence staining [9].

### 4.6. Transmission Electron Microscopy

A proportion of hippocampus and cortex was fixed in 2.5% glutaraldehyde solution, immersed in 1% osmium tetroxide at 4 °C, and then washed 3 times with 0.1 M PB. After fixation, the following steps were performed using an EM processor with agitation at room temperature. The tissues were dehydrated in graded ethanol (30%, 50%, 70%, 80%, 90%, 95% ethanol) for 5 min, each followed by three changes of absolute ethanol (each 10 min). After two changes in 1, 2-propylene oxide (PO) (15 min each), tissues were immersed in a 1:1 PO:Epon mixture for 1 h. These tissues were then incubated overnight in pure Epon and embedded in pure Epon at 60 C for 3 days. The Epon-embedded tissues were cut into 90 nm sections with a diamond knife on an ultracut microtome and collected on a 200 mesh copper grid. Lead citrate (approximately 3%) and 8% uranyl acetate were filtered before use. The grids were stained with lead citrate droplets for 20 min in a Petri dish, washed 3 times with distilled water, and were then ready for electron microscopic analysis [7,9].

### 4.7. Evans Blue (EB) Assay

The vascular permeability of the BBB was analyzed using the modified EB extravasation method (*n* = 5 per group) [9]. Rats were anesthetized with 60 mg/kg sodium pentobarbital and infused with EB dye (4 mL/kg; 2% in 0.9% normal saline) via the right femoral vein at 37 °C for 5 min. Two hours later, blood vessels were perfused with 300 mL of saline, and the brain was harvested. The EB-stained tissue sections (20 μm) were observed under an ultraviolet light filter using red laser excitation. Red staining indicated high vascular permeability in tissues. The staining intensity was quantified using ImageJ (NIH, Bethesda, MD, USA). One-half of the tissues were homogenized with 750 µL of N, N-dimethylformamide (Sigma, St. Louis, MO, USA). The tissue homogenates were maintained in the dark for 72 h at room temperature, centrifuged at 10,000× *g* for 25 min, and then analyzed using a spectrophotometer (Molecular Devices OptiMax, San Jose, CA, USA) at 610 nm. The dye concentration was expressed as µg/g of tissue weight [7].

### 4.8. Immunofluorescence Staining

Slides were warmed for 20 min on a slide warmer. A ring of wax was applied around the sections with a PAP pen (Invitrogen, Carlsbad, CA, USA). After being rinsed with 0.01 M Tris-buffered saline (TBS) for 10 min, the sections were permeabilized and blocked with 0.3% Triton X-100/10% normal goat serum in 0.01 M PBS for 30 min. Tissue sections were stained with primary polyclonal rabbit antibodies against phosphorylated tau (1:200; Thermo Fisher Scientific, Waltham, MA, USA), nuclear factor kappa B (NF-κB; 1:500; R&D Systems, Millipore, Billerica, MA, USA), CD68/ED1 (1:200; Santa Cruz Biotechnology, Santa Cruz, CA, USA), zonula occludens 1 (ZO-1,1:500; Santa Cruz Biotechnology, Santa Cruz, CA, USA), cyclooxygenase-2 (COX-2; 1:1000; Neuromics, Edina, MN, USA), synaptophysin (Syn; 1:1000; Thermo Fisher Scientific, Waltham, MA, USA), RhoA/Rho-kinase (1:300; Upstate Biotechnology, Lake Placid, NY, USA), intercellular cell adhesion molecule-1 (ICAM-1,1:200; Novus Biologicals, Centennial, CO, USA), angiotensinogen (AGT, 1:1000; Chemicon, Temecula, CA, USA), phospho-Tyr467 (PI3K/p85,1:200; Thermo Fisher Scientific, Waltham, MA, USA), Microtubule-associated protein 1A/1B-light chain 3 beta II (LC3BII,1:400; Novus Biologicals, Centennial, CO, USA), signal transducers and activators of transcription 3 (STAT3,1:500; Cayman Chemical, Ann Arbor, MI, USA), phosphorylated AKT (p-AKT; 1:500; Abcam, Cambridge, MA, USA), acetylcholine (ACH; 1:100; St. Louis, MO, USA), anti-caspase 3 (1:500; Cayman Chemical, Ann Arbor, MI, USA) and choline acetyltransferase (CHAT; 1:500; Abcam, Waltham, MA, USA) overnight at 4 °C. After being rinsed with PBS three times, sections were stained with a goat anti-rabbit/mouse IgG secondary antibody (1:200; Invitrogen, Carlsbad, CA, USA) at 37 °C for 1 h and mounted with Gel Mount antifade aqueous mounting medium (Southern Biotech, Birmingham, AL, USA). Primary antibody omission controls were used in order to further confirm the specificity of the immunohistochemical labeling. For all histological and immunohistochemical analyses, five transverse sections from each animal were randomly selected, and digital photomicrographs were obtained from three visual fields per section. Micrographs of three visual fields on each section were taken at 200× magnification. The quantification of the histological results was performed by an investigator that was blinded to the treatments. The Zo-1, CD34 syn, and ICAM-1-immunoreactive areas were calculated using the NIH ImageJ software. Cells expressing NF-κB, CD68, COX-2, RhoA, LC3BII, AGT, CHAT, ACH, STAT3, tau, p-AKT, caspase-3, and PI3K/p85 in each image were counted.

### 4.9. Enzyme-Linked Immunosorbent Assay (ELISA)

Rats were sacrificed by decapitation at 4 weeks post-induction. Peripheral blood samples were collected (*n* = 3 per group per time point) at 4 °C. The blood samples were centrifuged at 1000× *g* for 20 min and then at 10,000× *g* at 4 °C for 10 min before storage. To measure the expression levels of cytokines, plasma samples were added to 96-well plates pre-coated with anti-interleukin-1 (IL-1β; BioLegend Inc. San Diego, CA, USA) primary antibodies for 1 h at 37 °C. Then, samples were incubated with a goat anti-rabbit IgG secondary antibody (1:2000; Bio-Rad, Hercules, CA, USA) for 60 min at 37 °C. The optical density of protein bands at 450 nm was measured using a Model 680 Microplate Reader (Bio-Rad), and the results were analyzed using GraphPad Prism (version 4; GraphPad, San Diego, CA, USA).

### 4.10. Western Blot

Rats were sacrificed by decapitation at 4 weeks post-induction (*n* = 4 per group per time point). The whole brain was collected from each animal. Proteins were separated by 12% SDS-PAGE electrophoresis and blotted onto polyvinylidene difluoride membranes. The membranes were then incubated with primary polyclonal rabbit antibodies against phosphorylated tau (p-tau; 1:400; Thermo Fisher Scientific), LC3BII (1:800; Novus Biologicals), NF-κB (1:800; R&D Systems), CD68/ED1 (1:500; Santa Cruz Biotechnology), ZO-1 (1:800; Santa Cruz Biotechnology), COX-2 (1:1500; Neuromics, Edina, MN, USA), Syn (1:1200; Thermo Fisher Scientific), RhoA (1:500; Upstate Biotechnology), ICAM-1 (1:500; Novus Biologicals), AGT (1:1200; Chemicon), PI3K/p85 (1:500; Thermo Fisher Scientific), STAT3 (1:800; Cayman Chemical), p-AKT (1:800; Abcam), ACH (1:500; Sigma), caspase 3 (1:1000; Cayman Chemical), and CHAT (1:500; Abcam) at room temperature for 12 h. The membranes were then re-probed with a rabbit anti-β-actin antibody (1:5000; Abcam). After incubation with a goat anti-rabbit secondary antibody (1:5000; Santa Cruz), the protein bands were detected using an enhanced chemiluminescence reagent.

### 4.11. Statistical Analysis

Data are presented as mean ± standard deviation (SD) unless otherwise indicated. All data were analyzed using the SPSS 13.0 software. Differences between protein levels were analyzed by a two-way analysis of variance (ANOVA) followed by Tukey’s post hoc test. Differences between histological scores were analyzed using a Mann–Whitney U test. The Kruskal–Wallis nonparametric one-way ANOVA was used to compare data presented as percentages. A *p*-value of less than 0.05 was considered statistically significant. All graphs were plotted using GraphPad Prism Version 4.0. Statistical analysis was also performed by a statistician that was blinded to the study design.

## 5. Conclusions

In conclusion, treatment with C16 plus Ang-1 alleviated functional disability and reduced neuronal death in rat brain cortex and hippocampus by ameliorating inflammatory cell infiltration into the CNS, protecting endothelial cells of blood vessels, and maintaining BBB permeability. This treatment might exert an anti-MA effect by activating the PI3K/AKT pathway. The combined treatment with C16 plus Ang-1 can be considered as a potential therapeutic option for AD and warrants further investigation.

## Figures and Tables

**Table 1:** The escape latency of three experimental groups in the positioning navigation test.

	Sham Control	Vehicle	C + A
1 day	13.11 ± 2.15	101 ± 7.74 *	30.11 ± 3.82 *^&^
2 days	13.22 ± 3.23	74.11 ± 3.51 *	27.88 ± 5.34 *^&^
3 days	5.0 ± 1.73	43.1 ± 8.28 *	14.2 ± 1.56 *^&^
4 days	4.3 ± 1.8	47.78 ± 5.1 *	15 ± 2.18 *^&^
5 days	4.4 ± 1.74	47.67 ± 3.67 *	15.1 ± 1.76 *^&^
6 days	3.89 ± 1.16	44.89 ± 5.64 *	10.3 ± 2 *^&^

* *p* < 0.05 vs. sham control group, & *p* < 0.05 vs. vehicle group.

## Data Availability

Data is contained within the article and Supplementary Material.

## Supplementary Materials

The following are available online at https://www.mdpi.com/article/10.3390/ph15040471/s1, Supplementary Figure S1: (A–F): Immunofluorescence staining (green) of macrophage-specific marker CD68 in the hippocampus of all three experimental groups. (G) Quantification of CD68-positive cells showed that treatment with C16 plus Ang-1 downregulated CD68 in AD rats. Scale bar = 100 µm. a, *p* < 0.05 vs. sham control group; b, *p* < 0.05 vs. vehicle group. Supplementary Figure S2: (A–H): Immunofluorescence staining of caspase-3 showed apoptotic cells (red) in all three experimental groups. The arrow in the high magnification images in C and G demonstrated the expression of caspase-3 in the cytoplasm. The cell nuclei in the motor cortex and hippocampus were visualized by Hoechst 33342 staining (blue). (I) Quantification of caspase-3-labeled nuclei showed that treatment with C16 plus Ang-1 reduced apoptotic cell death in AD rats. Scale bar = 100 µm. (J) Western blot analysis of the expression of apoptotic marker caspase-3. (K) Semi-quantitative profiles of protein bands showed that C16 plus Ang-1 suppressed the upregulation of caspase-3 in the vehicle group. caspase-3, 31 KD; β-actin, 42 KD. a, *p* < 0.05 vs. sham control group; b, *p* < 0.05 vs. vehicle group. Supplementary Figure S3: (A–I): Immunofluorescence staining (green) of RhoA/Rho kinase (RHOA) in the motor cortex and hippocampus. (J) Quantification of RHO-positive cells showed that treatment with C16 plus Ang-1 reduced the expression of RHO in AD rats. Scale bar = 100 µm. a, *p* < 0.05 vs. sham control group; b, *p* < 0.05 vs. vehicle group. Supplementary Figure S4: (A–I): Immunofluorescence staining (red) of angiotensinogen (AGT) in the motor cortex and hippocampus. The cell nuclei were visualized by Hoechst 33342 staining (blue). (J) Quantification of AGT-positive cells showed that treatment with C16 plus Ang-1 reduced the expression of AGT in AD rats. Scale bar = 100 µm. a, *p* < 0.05 vs. sham control group; b, *p* < 0.05 vs. vehicle group. Supplementary Figure S5: (A–I): Immunofluorescence staining (green) of activators of transcription 3 (STAT3) in the motor cortex and hippocampus. (J) Quantification of STAT3-positive cells showed that treatment with C16 plus Ang-1 reduced the expression of STAT3 in AD rats. Scale bar = 100 µm. a, *p* < 0.05 vs. sham control group; b, *p* < 0.05 vs. vehicle group. Supplementary Figure S6: (A–F): Immunofluorescence staining (green) of phosphorylated AKT (p-AKT) in the motor cortex and hippocampus. (H-M): Immunofluorescence staining (green) of PI3K in the motor cortex and hippocampus. (G, N) Quantification of p-AKT- and PI3K-positive cells showed that treatment with C16 plus Ang-1 increased the expressions of p-AKT (G) and PI3K (N) in AD rats. Scale bar = 100 µm. a, *p* < 0.05 vs. sham control group; b, *p* < 0.05 vs. vehicle group. Supplementary Figure S7: The flowchart o drug administration in the different groups.
