# Correction: Fu et al. C16 Peptide and Ang-1 Improve Functional Disability and Pathological Changes in an Alzheimer’s Disease Model Associated with Vascular Dysfunction. *Pharmaceuticals* 2022, *15*, 471

**DOI:** 10.3390/ph18070985

**Published:** 2025-06-30

**Authors:** Xiaoxiao Fu, Jing Wang, Huaying Cai, Hong Jiang, Shu Han

**Affiliations:** 1 Institute of Anatomy, Medical College, Zhejiang University, Hangzhou 310058, China; 21818569@zju.edu.cn; 2 Department of Neurology, Sir Run Run Shaw Hospital, Medical College, Zhejiang University, Hangzhou 310058, China; wangjinjoy@zju.edu.cn (J.W.); caihuaying2004@zju.edu.cn (H.C.); jianghong1975@zju.edu.cn (H.J.)

## Error in Figure

In the original publication [1], there was a mistake in Figure 7 as published. In Figure 7, the magnification of subfigure D,H,L is different from other subfigures. The corrected Figure 7 appears below. The authors state that the scientific conclusions are unaffected. This correction was approved by the Academic Editor. The original publication has also been updated.

## Figures and Tables

**Figure 7:**
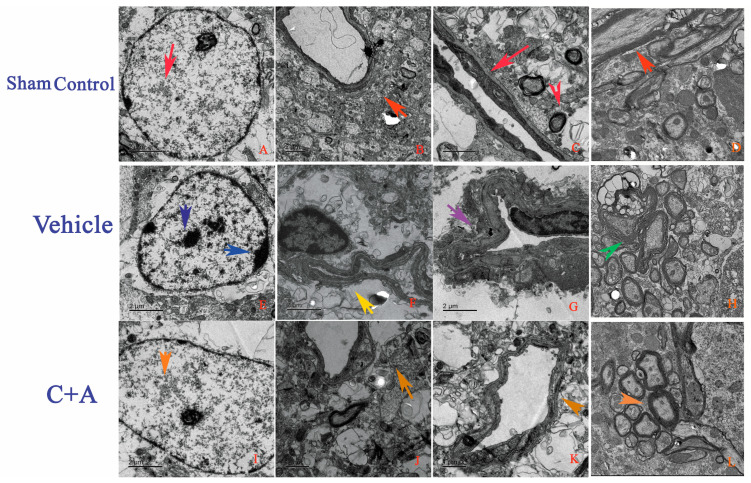
Electron micrographs show the ultrastructural morphology of the (**A**–**D**) sham control, (**E**–**H**) vehicle, and (**I**–**L**) C + A groups. The sham control rats showed (**A**) neuronal nuclei with uncondensed chromatin (red arrow in (**A**)). There was (**B**) no tissue edema or blood vessel leakage (red arrow in (**B**)). Intact tight junctions (red arrow in (**C**)) and myelinated axons surrounded by dark, ring-shaped myelin sheaths were also observed (red arrows in (**C**,**D**)). In the vehicle group, (**E**) neuronal apoptosis was evidenced by shrunken nuclei with marginated, fragmented, and condensed nuclear chromatin (showed by two arrows in (**E**)). (**F**) Severe blood vessel leakage and tissue edema in the extracellular space surrounding the vessels (yellow arrow in (**F**)). (**G**) Loosening of tight junctions between the endothelium (purple arrow in (**G**)). (**H**) Myelin sheath loosening and splitting (green arrow in (**H**)). Treatment with C16 plus Ang-1 reduced morphological changes of the nuclei (orange arrow in (**I**)), alleviated perivascular edema (orange arrow in (**J**)), prevented destruction of tight junctions (orange arrow in (**K**)), and decreased myelin sheath splitting (orange arrow in (**L**)).
