# Supplementary figures and images for: C16 Peptide and Ang-1 Improve Functional Disability and Pathological Changes in an Alzheimer’s Disease Model Associated with Vascular Dysfunction
Source: Pharmaceuticals (Basel). 2022 Apr 13;15(4):471. doi: 10.3390/ph15040471 (PMC9025163; doi:10.3390/ph15040471)

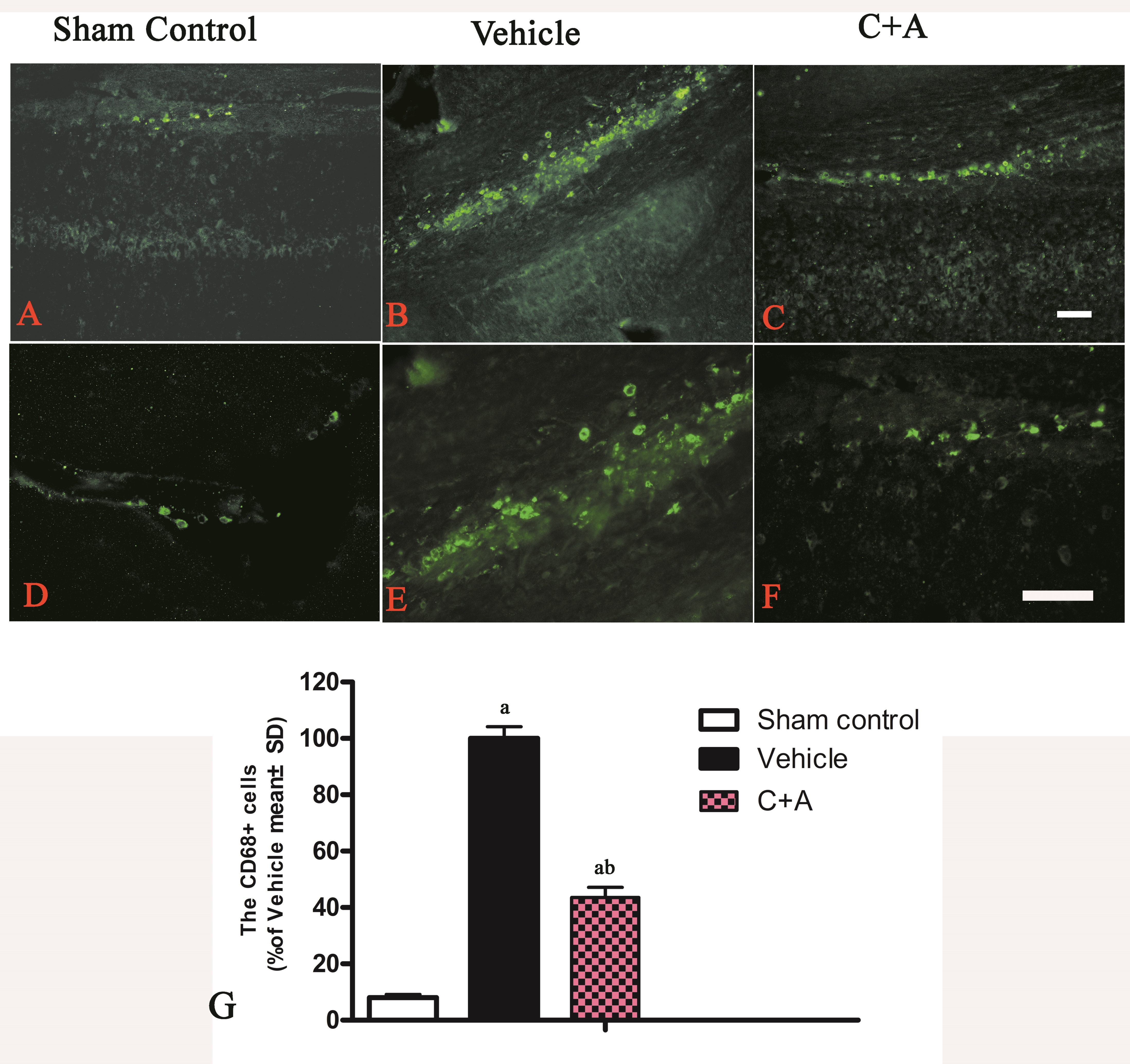

Supplement: Supplementary file 1 [file pharmaceuticals-15-00471-s001.zip › Supple Figure S1.jpg]

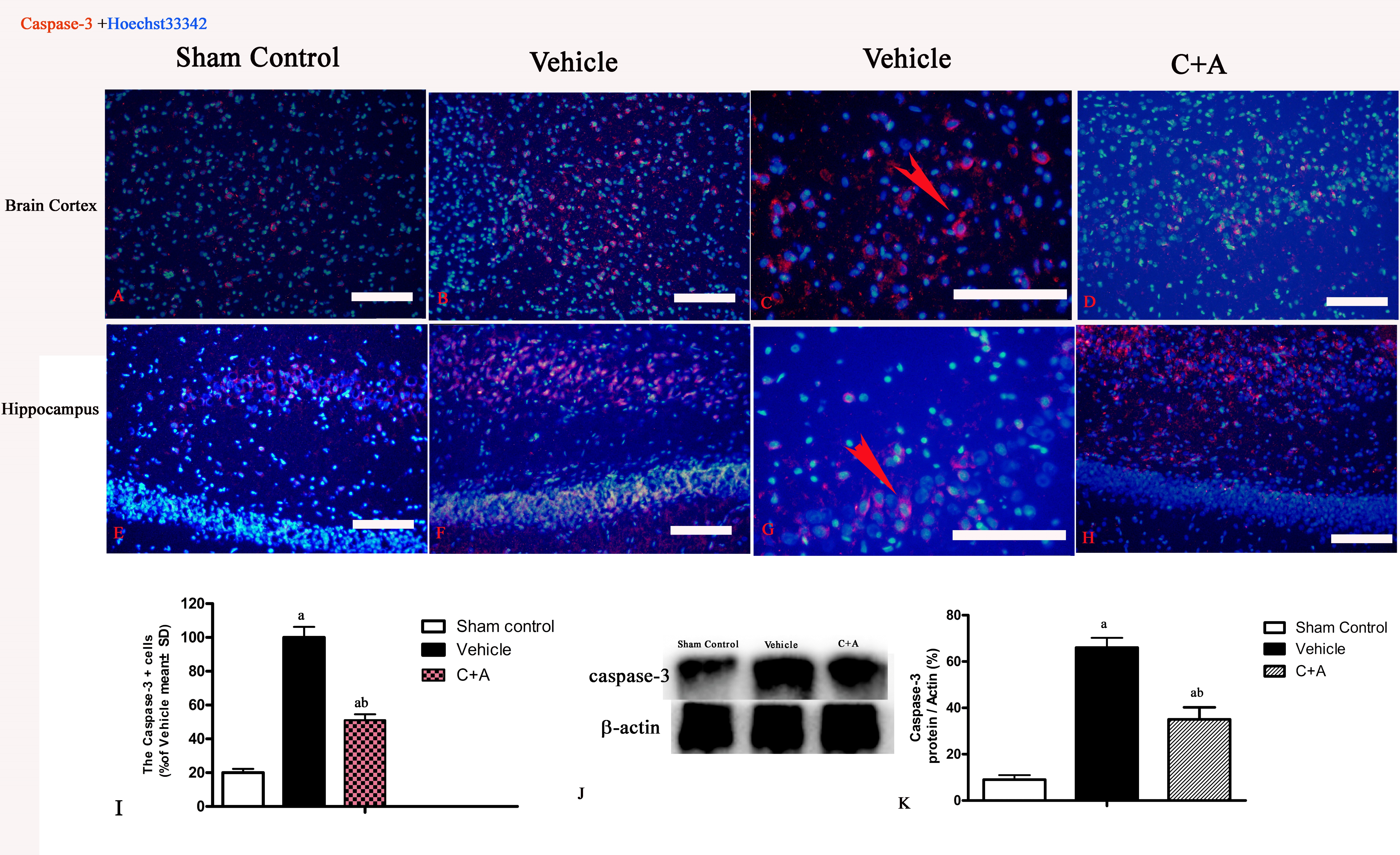

Supplement: Supplementary file 1 [file pharmaceuticals-15-00471-s001.zip › Supple Figure S2.jpg]

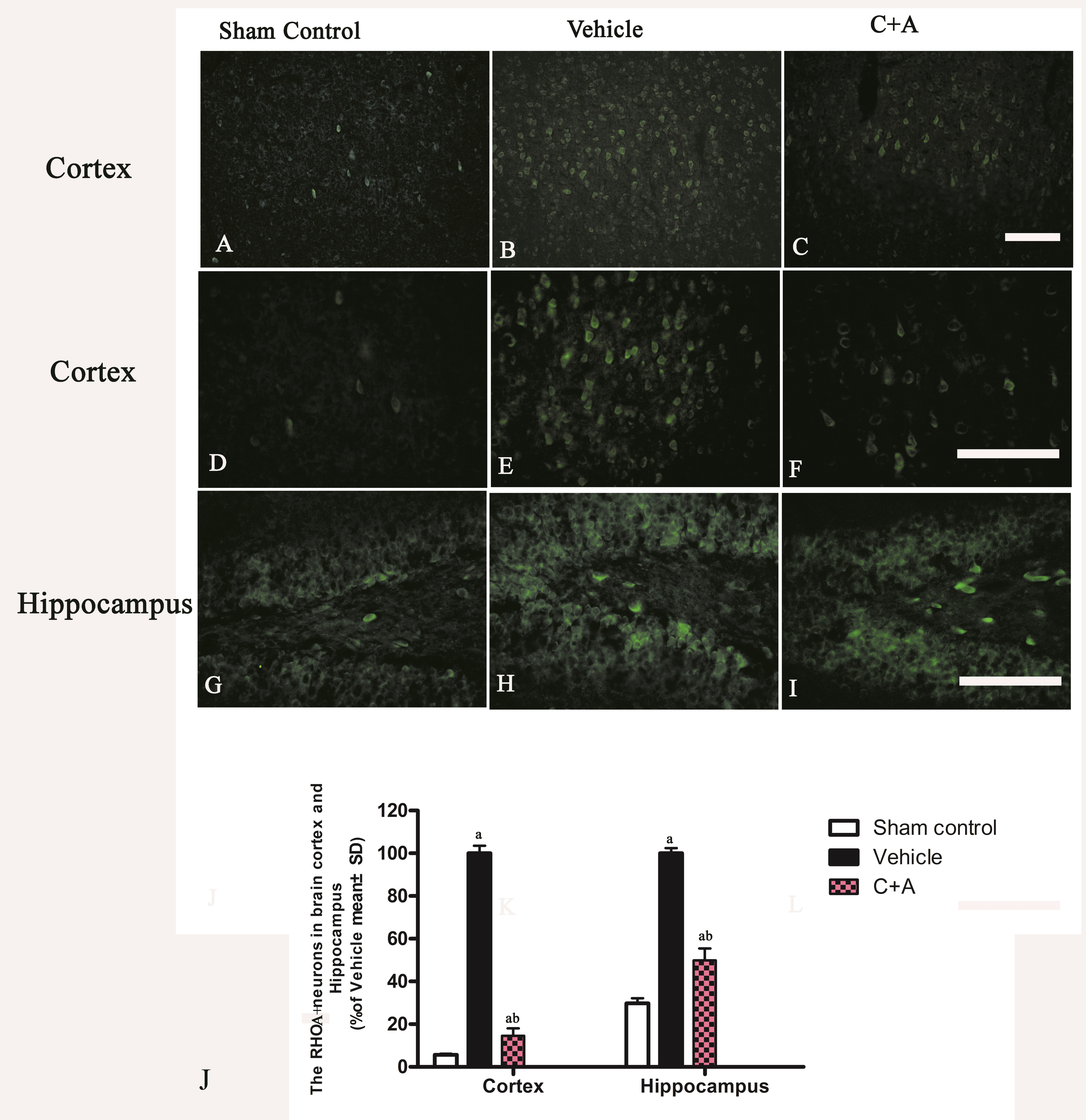

Supplement: Supplementary file 1 [file pharmaceuticals-15-00471-s001.zip › supple Figure S3.jpg]

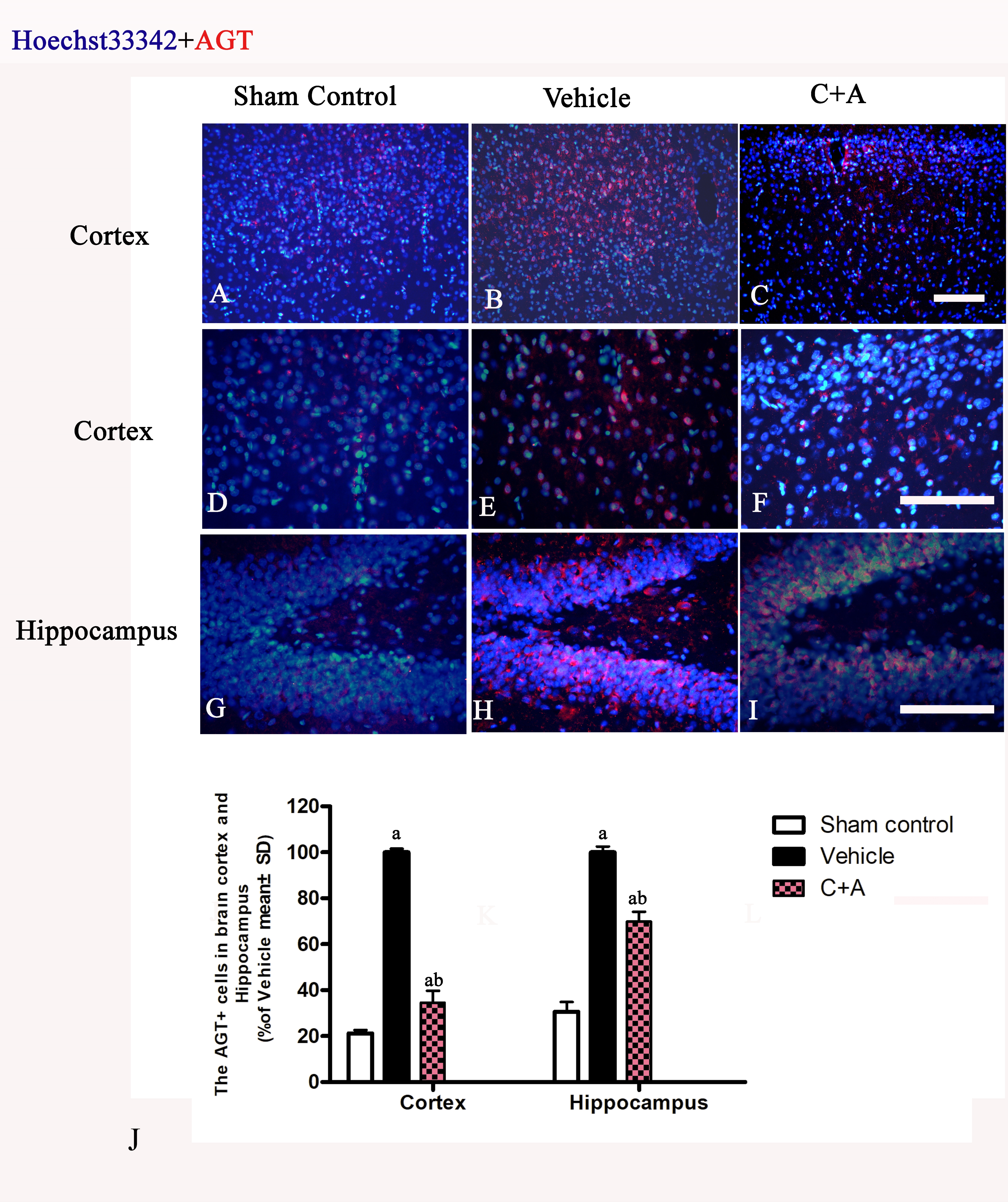

Supplement: Supplementary file 1 [file pharmaceuticals-15-00471-s001.zip › Supple Figure S4.jpg]

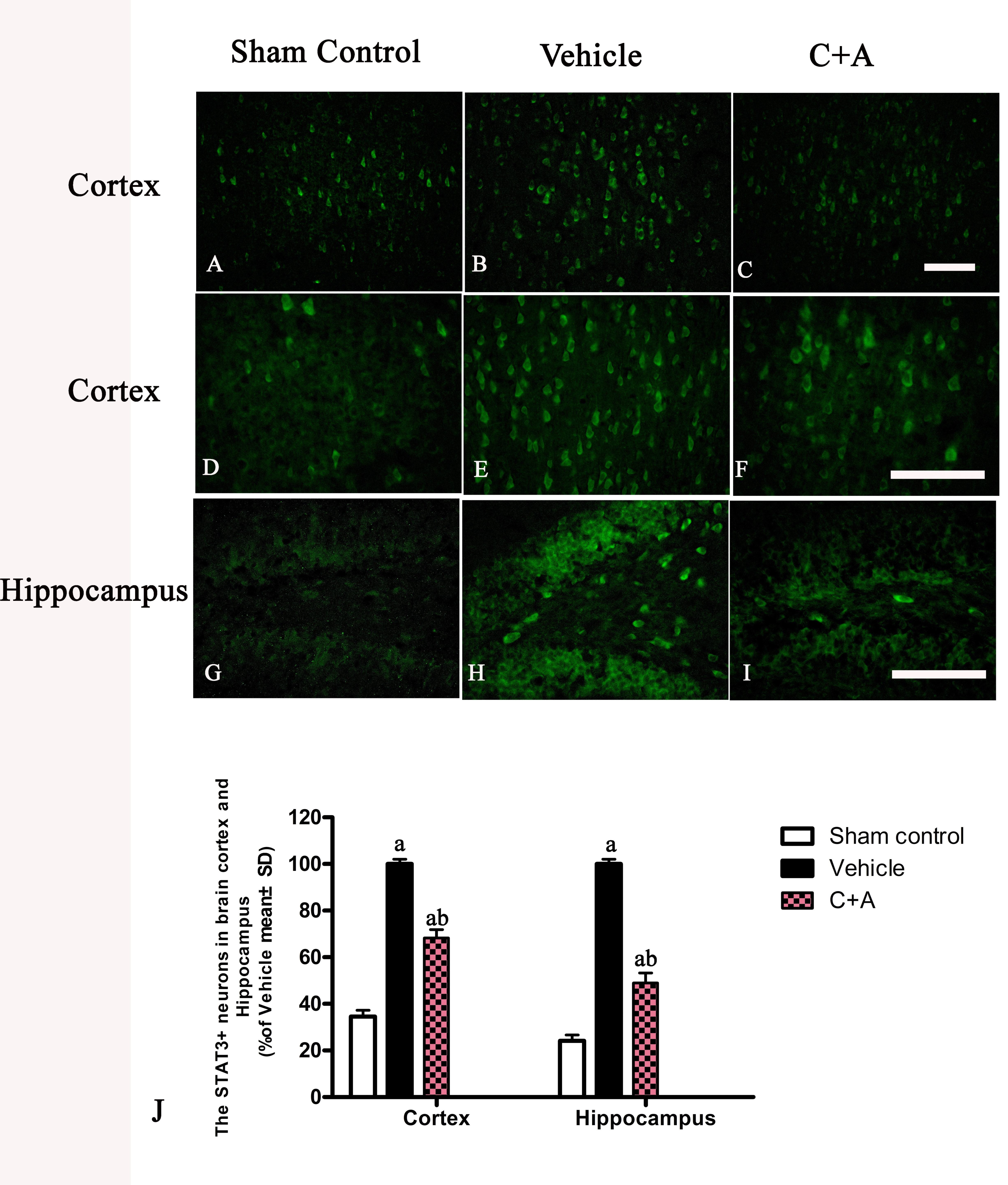

Supplement: Supplementary file 1 [file pharmaceuticals-15-00471-s001.zip › Supple Figure S5.jpg]

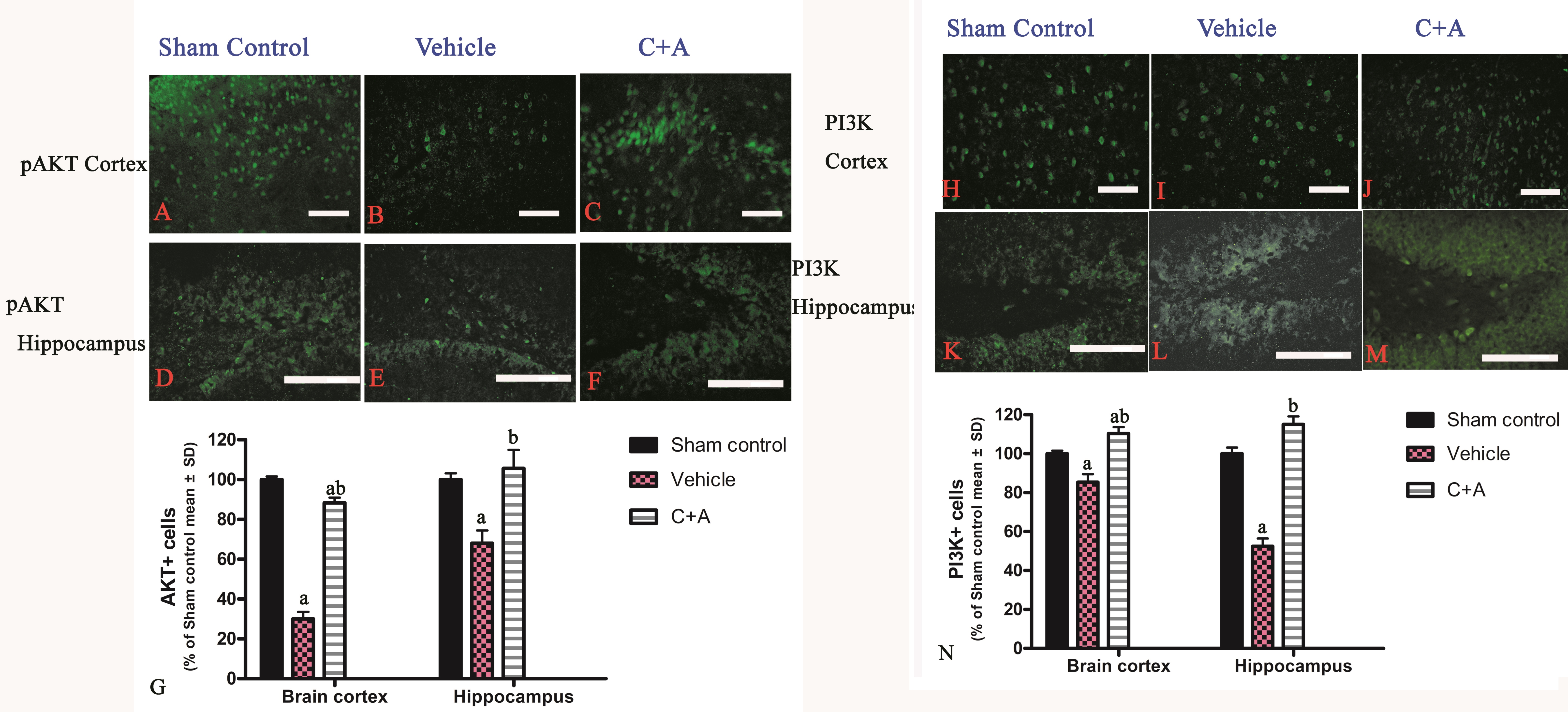

Supplement: Supplementary file 1 [file pharmaceuticals-15-00471-s001.zip › Supple Figure S6.jpg]

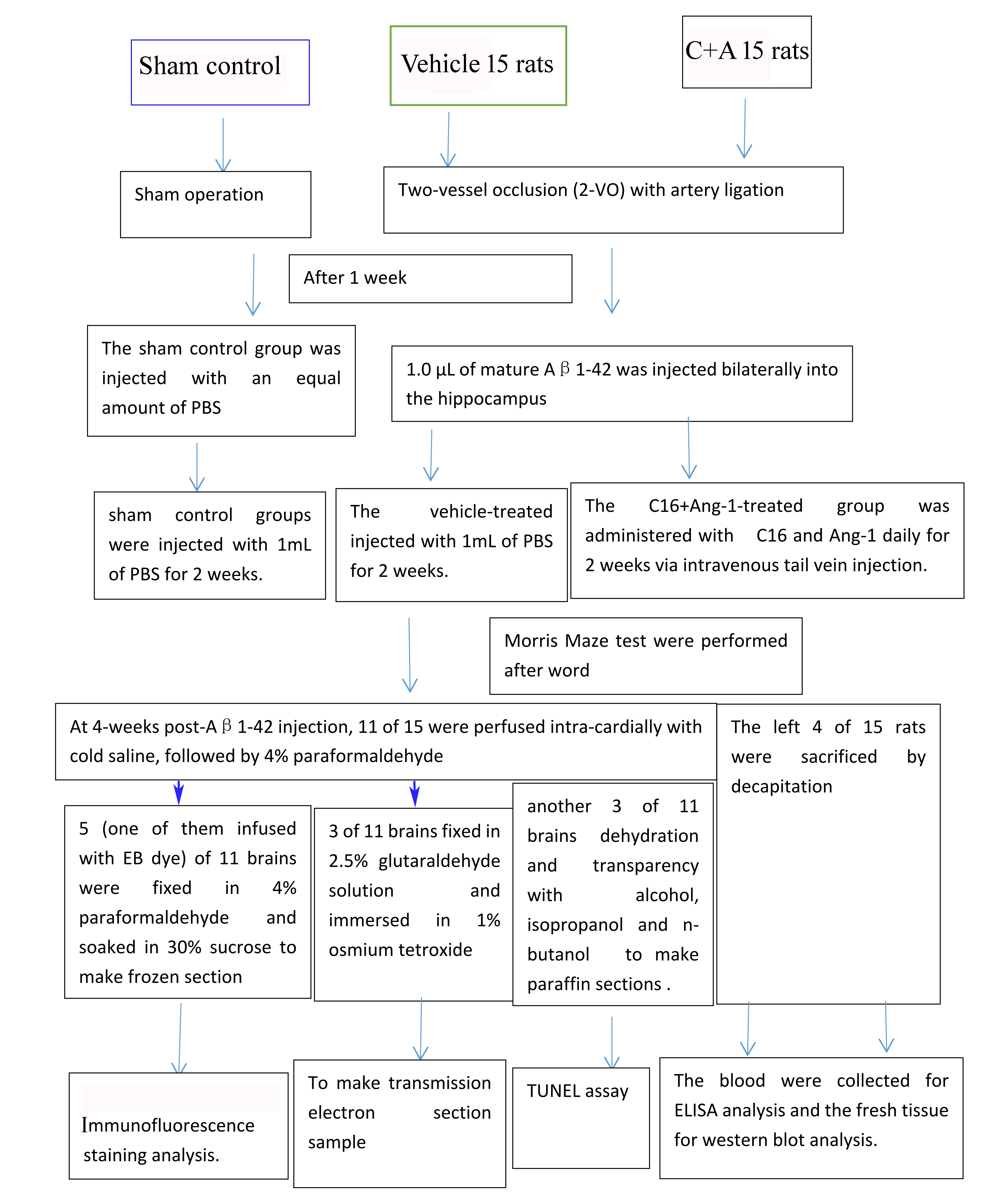

Supplement: Supplementary file 1 [file pharmaceuticals-15-00471-s001.zip › Supple Figure S7.jpg]
